# The roles of endolithic fungi in bioerosion and disease in marine ecosystems. II. Potential facultatively parasitic anamorphic ascomycetes can cause disease in corals and molluscs

**DOI:** 10.1080/21501203.2017.1371802

**Published:** 2017-08-31

**Authors:** Frank H. Gleason, Geoffrey M. Gadd, John I. Pitt, Anthony W. D. Larkum

**Affiliations:** a School of Life and Environmental Sciences, University of Sydney, Sydney, Australia; b Geomicrobiology Group, School of Life Sciences, University of Dundee, Dundee, UK; c Food, Safety and Quality, CSIRO, Ryde, Australia

**Keywords:** Calcareous substrates, calcium carbonate, shell disease, eutrophication, temperature, pH

## Abstract

Anamorphic ascomycetes have been implicated as causative agents of diseases in tissues and skeletons of hard corals, in tissues of soft corals (sea fans) and in tissues and shells of molluscs. Opportunist marine fungal pathogens, such as *Aspergillus sydowii*, are important components of marine mycoplankton and are ubiquitous in the open oceans, intertidal zones and marine sediments. These fungi can cause infection in or at least can be associated with animals which live in these ecosystems. *A. sydowii* can produce toxins which inhibit photosynthesis in and the growth of coral zooxanthellae. The prevalence of many documented infections has increased in frequency and severity in recent decades with the changing impacts of physical and chemical factors, such as temperature, acidity and eutrophication. Changes in these factors are thought to cause significant loss of biodiversity in marine ecosystems on a global scale in general, and especially in coral reefs and shallow bays.

## Introduction

### Primary objectives of this review

Many species of fungi are known to bore into solid rock, sand grains and shells and can cause disease in many animal and plant tissues in marine ecosystems (Rosenberg and Ben-Haim 2002; Golubic et al. 2005; Pitt and Hocking 2009; Raghukumar and Ravindran 2012; Burge et al. 2013). In this review, we discuss what is known about the different ecotypes of Ascomycota (true fungi) with emphasis on anamorphic stages of *Aspergillus sydowii* and other species, which bore into skeletons of corals or shells of molluscs and infect the soft tissues of these animals causing diseases in marine ecosystems. The sexual or teleomorphic stage of *A. sydowii* is *Emericella sydowii*.

We briefly describe some key aspects of their morphology, life history, mechanisms of infection, general roles in ecology, host substrate interactions, participation in the marine calcium carbonate cycle, and the possible effects of global climate change factors on growth wherever possible. However, there is not enough relevant data available to adequately understand the roles of fungi in diseases of corals and molluscs in marine ecosystems. In fact, there is little known about the ecological roles of fungi in aquatic ecosystems in general. We assume that fungi are usually saprotrophs and sometimes parasites, but the potential roles of many fungi could be beneficial to their hosts in symbioses including the examples mentioned here. More research is required to determine the specific ecological role of each species.

### Dust storms pollute the ocean and cause growth of fungi

Many species of Ascomycota with airborne spores (such as species of *Aspergillus, Penicillium, Cladosporium, Fusarium* and *Alternaria*) commonly grow on many organic substrates in nutritionally rich environments. The substrates include living and dead plant and animal hosts, body parts and detritus in soils, sands and sediments (Burge et al. 2013). When the substrates dry out, fungi characteristically form conidia and release clouds of spores into the air, which can be significant components of dust storms formed in deserts such as in Africa (Weir-Brush et al. 2004) and in Australia (Hallegraeff et al. 2014). Dust storms can therefore be an important mechanism for dispersal of spores from terrestrial into aquatic environments. Formation of conidia and dispersal by currents can occur in both fresh and salt water environments as well, but drying is necessary to get the spores airborne.

Although these species are essentially terrestrial fungi, they have adapted to the higher salinities found in the ocean. Spores of these fungi often germinate easily, and the growth patterns of these fungi cause the formation of fungal blooms which can include floating rafts of hyphae in coastal marine ecosystems (Hallegraeff et al. 2014), especially with high nutrient content (Zverev and Vysotskaya 2005), i.e. high concentrations of utilisable organic compounds dissolved in water, formed during the process of eutrophication. These fungi can grow within a wide range of temperatures, pH values and salinities (Parekh and Chhatpar 1989; Sharma et al. 2011) which suggests that the rate of growth of these fungi might increase with increases in temperature and acidity in the ocean as a result of global climate change. The effects of temperature, salinity, pH and other physical factors on the growth of marine Ascomycetes are discussed later.

## Other mechanisms for dispersal

It is possible that marine animals can be potential reservoirs for survival of pathogens and play a role in their dispersal. For example, Ein-Gil et al. (2009) documented *A. sydowii* growing in tissues of healthy sponges (*Spongia obscura*) collected from mangrove roots along a pristine tidal channel at Grand Bahama Island. They proposed that this sponge may be a symptomless carrier of this pathogen which can cause disease in gorgonian corals. Fungal spores can be released after growth and conidiation in live sponges or after their death and hopping by live sponges may result in dispersal of fungal spores.

## Fungal pathogens in marine ecosystems

Many opportunistic marine fungal pathogens such as anamorphic species of Ascomycota rapidly produce large numbers of conidia (air- and water-borne spores) asexually (r or ruderal strategy) (Pitt and Hocking 2009) and are ubiquitous in the open oceans, intertidal zones, marine sediments and animals, which live in these ecosystems (Burge et al. 2013). Under certain conditions, the teleomorphic species of Ascomycota reproduce sexually (s-strategy) to form ascospores in cleistothecia, perithecia or apothecia sometimes in large fruiting bodies (Pitt and Hocking 2009). Fruiting bodies have not been observed for many species in the ocean, which makes identification more difficult. The prevalence and consequences of infection caused by Ascomycota and other fungi have increased in frequency and severity in recent decades (Soler-Hurtado et al. 2016). Many species of Ascomycota produce toxins, which are directly harmful to many animals including humans. These toxins accumulate in food webs and poison food supplies and drinking water (Pitt and Hocking 2009; Burge et al. 2013).

## Fungal diseases of corals

### A. sydowii, a putative causative agent of aspergillosis in sea fans (soft corals)

Burge et al. (2013) and Hayashi et al. (2016) found many species of anamorphous Ascomycetes associated with corals in the Caribbean and the South Pacific. Their data suggest that *A. sydowii* is the causative agent of aspergillosis in the gorgonian soft corals, *Gorginia flaballum* and *Gorgonia ventalina*. However, Toledo-Hernández et al. (2008) stated that they doubted that *A. sydowii* is the only fungus that can cause aspergillosis in *G. ventalina* because it is not always present in diseased corals. Soler-Hurtado et al. (2016) found *A. sydowii* and other potential fungal pathogens in gorgonian octocorals of Ecuador (see below). Since *A. sydowii* can be present in healthy corals, perhaps environmental factors are involved as well. We have selected *A. sydowii* as an example of an emerging parasitic fungus because it has been studied more frequently and more is known about it than the other species (Table 1). The key characteristics of this species listed in Table 1 are based on the papers cited here. [More information is available in Pitt and Hocking (2009) and Alker et al. (2001).] A list of many species of fungi isolated from tissues of octocorals in two recent studies is given in Table 2.

**Table 1.** Characteristics of the potential emerging facultative parasite, *Aspergillus sydowii.*

Category	Characteristic	Range	Optimum	Reference
Physical	Temperature	0–37°C	30–35°C	Alker et al. (2001)
		>40°C		Sharma et al. (2011)
	pH	pH 4–9	pH 7	Sharma et al. (2011)
	Salinity	Soil and fresh water to full strength sea water	ND	Hayashi et al. (2016)
		Soil, seawater and salt solutions up to 20 ppm (w/w)	ND	Pitt and Hocking (2009)
	Nutrient concentration	Very low to very high, oligotrophic to eutrophic	ND	
Biological	Host range	Wide, including a few angiosperms, invertebrates and vertebrates, but poorly studied	Burge et al. (2013)	
	Asexual dispersal unit	Conidia, >5 µm	Hallegraeff et al. (2014)	
		Conidia, 2.5–3 5 um	Pitt and Hocking (2009)	
	Sexual dispersal unit	Ascospores in Cleistothecia in some isolates of *Aspergillus*, but yet not determined for *A. sydowii*	Hallegraeff et al. (2014)	
	Dominant ecological strategies	Mostly r and sometimes s strategies		
	Toxins	Yes, but uncharacterised	Hayashi et al. (2016)	
	Secondary metabolites	Sydowinol, sydowinins, hydroxysydonic acid	Hayashi et al. (2016)	
Excreted extracellular enzymes for substrate digestion	Proteases	Serine protease for cell membranes of animal cells	Sharma et al. (2011)	
	Cellulases	β-Endoglucosidases for plant cell walls	Matkar et al. (2013)	
	Xylanases	β-Xylanases for plant cell walls	Ghosh et al. (1993)	

This fungus is well adapted to a wide range of environmental conditions and its prevalence is rapidly increasing in animal hosts which are presently being studied. ND: Not detected.

**Table 2.** Some species of fungi isolated from tissues of octocorals.

Genus	Species	Host	Location	References
*Alternaria*	sp.	*Leptogorgia* sp and *L. obscura* (octocorals)	Ecuador	Soler-Hurtado et al. (2016)
*Aspergillus*	*ochraceopetaliformis*			
	*sclerotiorum*			
	*sydowii*			
	*wentii*			
*Capriobotryella*	sp.			
*Cladosporium*	dominicarium			
	sphaerospermium			
*Curvularia*	sp.			
*Fusarium*	*longipes*			
*Lasiodiplodia*	*pseudotheobromae*			
*Nigrospora*	sp.			
*Penicillium*	*chrysogenium*			
	*mallochi*			
*Phoma*	sp.			
*Pyrenochaetopsis*	*leptospora*			
*Tritirachium*	sp.			
*Acremonium*	sp.	*Pacifigorgia* and other octocorals	Columbia	Barrero-Canosa et al. (2013)
*Amaurodon*	sp.			
*Aspergillus*	*versicolor*			
	*sclerotiorum*			
	*sydowii*			
*Chaetomium*	*globosum*			
*Diaporthe*	*lusitianicae*			
*Earliella*	*scabrosa*			
*Endomelanconiopsis*	*endophytica*			
*Eutypella*	*scoparia*			
	sp.			
*Gliomastix*	*murorum*			
*Glomerella*	*cingulata*			
*Microdiplodia*	sp.			
*Penicilium*	*meleagrinium*			
	*steckii*			
	*sumatrense*			
*Xylaria*	*mali*			
	sp.			

Widespread death of soft corals, especially sea fans from the genus *Gorgonia*, in the Caribbean (Smith et al. 1996) and Florida Keys (Bruno et al. 2011) has been reported. The major species of pathogen involved in the Caribbean outbreak was subsequently identified unequivocally as the common terrestrial species *A. sydowii* by Geiser et al. (1998). However, this disease was reproducible using isolates only from diseased corals, but not from other sources, suggesting the influence of unknown factors leading to pathogenicity.

Taxonomic studies have shown the presence of a wide range of fungi associated with healthy corals. Soler-Hurtado et al. (2016) isolated 17 fungal species, including *A. sydowii* (7% of isolates) from healthy corals in Columbia. However, the samples were taken from a depth of 10–15 m, where low oxygen availability would limit fungal growth. Most isolates were identified as species of *Penicillium, Aspergillus, Cladosporium, Nigrospora* and *Fusarium*, though identifications were based on limited information.

Fungal diseases of soft corals have been discussed in some detail by Burge et al. (2013). They reported the incidence of aspergillosis, an emerging fungal disease caused by *A. sydowii* in gorgonian corals (*G. ventalina* and *G. flaballum*) in the Caribbean. Soler-Hurtado et al. (2016) also reported this disease in gorgonian corals (*Leptogorgia obscura* and L. sp.) along the Pacific coast of Columbia. Some isolates of *A. sydowii* are known to synthesise a wide range of secondary metabolites with the potential to be toxic to hard corals. Hayashi et al. (2016) attributed aspergillosis of gorgonian corals to secondary metabolites which were toxic to the coral symbiont, the dinoflagellate *Symbiodinium.*

### Inhibition of photosynthesis in hard corals by fungal toxins

The possible role of secondary metabolites in disease caused by *A. sydowii* and some of the other fungi isolated by Hallegraeff et al. (2014) have been studied further by Hayashi et al. (2016). They exposed small numbers of cells from cultures of three clades of *Symbiodinium* to extracts and purified compounds from culture media after growth of *A. sydowii*. Sydowinin A, sydowinin B, sydowinol and sydowic acid, metabolites produced exclusively by *A. sydowii*, caused significant inhibitory effects on photosynthetic fluorescence yield (*F*_o_/*F*_m_) of cultures from two of the three clades of *Symbiodinium* studied. This was shown by exposing small cultures of three clades of *Symbiodinium* to natural extracts, CS156 [Clade C], CS73 [Clade A] and CS163. Natural extracts from CS156 and CS73, but not CS163, depressed *F*_v_/*F*_m_ levels in the cultures over a matter of days. This is a measure of the photosynthetic yield (of photosynthesis) in these samples and shows that there is an inhibitory effect on photosynthesis. Similar effects could be shown more convincingly with Sydowinin A, Sydowinin B, Sydowinol and Sydowic acid, which are natural products excreted by *A. sydowii*. These results demonstrate that both extracts from *A. sydowii* and natural products from *A. sydowii* have the capacity to inhibit the photosynthetic rate of zooxanthellae.

While much more work needs to be carried out, it is clear that *A. sydowii* could combine the inhibition of photosynthesis with the effects of summer sea surface temperature rise and to exacerbate coral bleaching (Hayashi et al. 2016).

### Band diseases of hard corals

There are many reports on the pathogens involved in band diseases of hard corals and these are usually attributed to bacteria, including cyanobacteria (Bourne et al. 2009). However, fungal pathogens are also involved: Priess et al. (2000) reported an *Aspergillus*-like species which was an endolithic fungus and which attacked the endolithic algae in the black bands of the skeleton of *Porites lutea*, suffering from black band disease.

Ravindran et al. (2001) studied fungi in both healthy and diseased corals (*P. lutea* and *Porites compressa*) from the Lakshadweep Islands in the Indian Ocean. These fungi grew inside the skeleton and around the coral polyps and ultimately penetrated the coral tissues. Some of the species were isolated into pure culture. The genera included species from *Fusarium, Aspergillus, Cladosporium, Chaetomium*, *Aureobasidium*, and *Curvularia*. The roles of these fungi in causing disease were not determined.

The prevalence of coral associated ascomycetes and other fungi (some basidiomycetes and unidentified species) was four times higher in diseased colonies of *Acropora formosa* colonies than in healthy colonies (Yarden et al. 2007). The fungi isolated included *Phoma* sp., *Humicola fuscoatra, Cladosporium* sp. and *Penicilium citrinuma* in brown band disease and *Phoma* sp., *Fusarium* sp., *Aureobasidium pullans* and *Alternaria* sp. in skeletal eroding disease. These fungi may be constitutive, and their abundance may be dependent on the health of the coral colonies and environmental factors. No evidence to suggest that these fungi are primary pathogens has been reported.

## Aetiology and effects of shell disease in molluscs

Shell disease begins when photosynthetic microborers first penetrate the shell of molluscs. For example, Ćurin et al. (2014) discuss the incidence of a damaging endolithic infestation by photosynthetic microboring organisms in the edible bivalve *Modiolus barbatus*. While the characterisation of these endolithic algae has not been done properly, they are likely to be *Ostreobium* species or allies (Gutner-Hoch and Fine 2011), which were first identified in mollusc shells (Bornet and Flahault 1889). Later, the heterotrophic microborers arrive and attack the photosynthetic microborers and the protein scaffolding inside the shell, and their continued growth further damages the shell with time (Jones and Pemberton 1987; Golubic et al. 2005). The first sign of oyster shell disease is the appearance of small white or black spots on the sub-nacreous layer of the oyster shell, indicative of being infested with an endolithic fungus (Cole 1950; Alderman and Jones 1967; Raghukumar and Lande 1988). Eventually, the mycelium spreads throughout the shell and gives rise to warts consisting of conchiolin and displaying strong discolouration. Deformation of shell edges and hinges may occur, and warts may later coalesce and affect the adductor muscle to such an extent that it prevents the closure of the valves (Galtsoff 1964). Chronic symptoms include marked stimulation of shell growth and formation of shell chambers. The most obvious symptoms of the disease are black, green or brown conchiolin warts, which detracts from the value of the oyster and can be disfiguring and unpleasant (Alderman and Jones 1967). Shell disease has caused serious damage in the Netherlands (Korringa 1951), France (Marteil 1954), United Kingdom (Cole 1950; Alderman and Jones 1971b) and the Black Sea (Pirkova and Demenko 2008).

Peharda et al. (2015) reported considerable variation in the degree of shell damage and extent of infestation caused by heterotropic endoliths in *Lithophaga lithophaga* (date mussel) shells collected at two environmentally different sites (differing in productivity) in the Adriatic Sea. The species of heterotrophs were identified from the traces in different layers of the shell and from SEM within organic lamellae. Peharda et al. (2015) found a positive relationship between productivity in the ecosystems and rates of bioerosion in mussel shells.

Zverev and Vysotskaya (2005) and Borzkh and Zvereva (2012, 2015) found large assemblages of filamentous true fungi associated with populations of four different commercially important species of bivalve molluscs in the sea of Japan: *Crenomytilus grayanus, Anadara broughtoni, Modiolus modiolus* and *Crassostreas gigas*). The compositions of the assemblages of the Ascomycota associated with these molluscs were similar to the compositions found in assemblages associated with species of corals in other parts of the world. Borzkh and Zvereva (2012) identified 17 species of filamentous Ascomycota isolated from internal organs of the Pacific giant oyster. These fungi belonged to six genera: *Aternaria, Aspergillus, Botrytis, Fusarium, Penicillium* and *Trichoderma*.

Greco et al. (2017) isolated *A. sydowii* from marine-bottom sediments, water samples and calcareous shells of bivalve molluscs (*Mytilus galloprovincialis*) during a survey of fungi in the Port of Genoa (Italy, Liguria Sea, Mediterranean Sea).

Apart from shell weakening and disfigurement, many fungi can produce powerful toxins that can affect the hosts they invade or build up in the mollusc tissues, becoming a problem for human consumption. While most studies available to date investigated fungi isolated from the soft tissues of molluscs, at least some of such toxic species are known to be microborers. According to Zverev and Vysotskaya (2005), the bulk of the ascomycetes and other fungi isolated from bivalve tissues in the Sea of Japan were pathogenic and toxigenic fungi. These included species of *Aspergillus, Penicillium, Cladosporium* and *Chaetomium*, which produce mycotoxins including aflatoxins, ochratoxins, gliotoxins and hemotoxins (Zverev and Vysotskaya 2005). These toxins partly determine the pathogenic properties of the fungi. Some of these fungi from both terrestrial and marine environments are known to cause mycotoxicosis in animals including humans (e.g. Sallenave et al. 1999; Grovel et al. 2003; Gulyás 2013).

Sallenave-Namont et al. (2000) isolated possible toxigenic saprotrophic fungi from samples of shellfish (*Cerastoderma edule, Mytilus edulis*), sediments and seawater from shellfish farming areas and from natural shellfish beds along the coast of France. Filtrates from the media used to grow the fungi were tested for their effects on larvae of the small crustacean *Artemia salinus*, which is highly sensitive to mycotoxins. 35.5 per cent of the 456 isolates including twelve genera actively produced mycotoxins (e.g. *Aspergillus, Penicillium, Trichoderma* and *Cladosporium*). Therefore, the study conducted by Sallenave-Namont et al. (2000) confirmed the existence of fungi producing mycotoxins in shellfish farming areas in France.

## Effects of the environment on diseases of corals and molluscs

### Eutrophication

As with many other bioeroders, eutrophication increases the diversity, abundance and bioerosion rates of fungi and fungus-like organisms. In this context, cnidarian-associated fungi are better investigated than those found on molluscs. Gorgonian aspergillosis increases with nutrient enrichment (Bruno et al. 2003). Black bands in corals were identified as concentrations of microborers, green algae and *Aspergillus*-like fungi (Bak and Laane 1987; Priess et al. 2000). The bands coincided with the end of the rainy season at the sample site, when build-ups of phototrophic microborers were followed by fungal invasions. It appears likely that this represents a chain of nutrient-related developments, with the rainwater washing nutrients in the water that fertilise the phototrophs, which then become the basis for fungal sustenance.

Zverev and Vysotskaya (2005) observed an abundant and diverse community of filamentous fungi in a marine environment polluted by industrial and municipal waste (Ussuriysk Bay, Sea of Japan). They isolated 155 strains of filamentous and sac fungi (Ascomycetes) from the internal tissues of two species of bivalves (*C. grayanus* and *M. modiolus*). This complex of filamentous and sac fungi included opportunistic parasites and represented species that are widely distributed in both terrestrial and marine habitats. The growth of most fungi is stimulated by the presence of utilisable carbohydrate sources and nutrients. Zvereva and Vysotskaya’s (2005) observations were undoubtedly caused by eutrophication which provided a large inoculum with many species for infecting bivalves.

Carreiro-Silva et al. (2009) conducted a semi-controlled experiment on microbioerosion in *Lobatus gigas* shell fragments under different modes of fertilisation. When organic nitrogen or phosphorus was added, bioerosion by photosynthetic microborers was enhanced. If organic matter was added by itself, heterotrophic microborers, chiefly fungi, became more dominant and colonised almost 50% of the substrate area and eroded more of the surface area.

#### Temperature

Boyd and Kohlmeyer (1982) found that the marine fungi they studied were best adapted to the temperatures in the climates in which they occurred, and temperature regimes determine their distributions (Kohlmeyer 1983). Nevertheless, fungi that are known to cause marine diseases can thrive in unusually high temperatures. One example of this is *A. sydowii* which causes asperillosis, a disease of *G. ventalina* (Ward et al. 2007). This disease has become epidemic in sea fan populations in the Caribbean with the warming of ocean temperatures in mid-summer to over 30°C. The optimum temperature for the growth of this parasite is 31°C. At 31°C, infected host tissues lost over 30% of their zooxanthellae as compared to 27°C. However, during the summer, the host resistance increased as well. Host resistance was measured by the inhibition of the growth rate of the fungal pathogen on agar plates by crude extracts from the host tissues. Another example is shell disease in molluscs. Early observations on shell disease caused by *Ostracoblabe implexa* were linked to the occurrence of a few weeks of above-normal temperatures (Korringa 1951; Alderman and Jones 1971b; Alderman 1976, 1985). Aspergillosis of benthic organisms was also commonly observed under conditions of heat stress (e.g. Cerrano et al. 2000; Perez et al. 2000).

It is not quite clear whether this indicates a heightened vulnerability of the hosts under heat stress or reflects fungal blooms at certain water temperatures. Fungi have certainly been observed to grow faster and to reproduce at temperature optima, which are often comparatively higher than that of the marine environment where they occur. Some marine fungi can be locally more diverse at higher temperatures (Amend et al. 2012). Other species appear to prefer colder environments (Wisshak and Porter 2006). As the surface of the ocean warms, coral bleaching is one of the first phenomena to be observed (see Gleason et al. 2017). It is widely predicted that coral bleaching will lead to the demise of corals and coral reefs.

Almost certainly, there will be major interactions between fungal parasites, coral substrates and defence reactions of the host animals. The facultative parasite of corals, *A. sydowii*, is involved in this interplay but the details need to be fully worked out in future research (Gleason et al. 2017). One preliminary study focused on the effects of temperature on the rate of secretion of protease enzymes produced by the pathogen, *A. sydowii*, and protease inhibitors secreted by the host, *G. ventalina* (Mann et al. 2014). At elevated temperatures (30°C), protease activity was significantly higher than at ambient temperatures (25°C) in *A. sydowii*. Temperature stress did not induce a change in protease inhibitor activity in *G. ventalina* colonies but protease inhibitor activity was always less in diseased than in healthy individuals at all temperatures tested.

### Ocean acidification

Where experiments were conducted on the growth of marine fungi, they usually grew best in slightly acidified media (e.g. Curran 1980). It also stands to reason that acidified water would shift the ratio between inorganic to organic materials in mollusc shells through shell dissolution towards the organic food sources of the fungi, which might support fungal growth.

Factor and organism interactions can at times complicate pattern recognition. Fungi responsible for coral lesions have been found to grow slower under reduced pH, which could slow down the temperature-induced progression of fungal diseases of corals (Williams et al. 2014). However, the authors suggest that the effect for bioeroding fungi would be similar as stated above: The effects of acidification on the skeletal properties of the host organism may favour faster degradation of the skeleton. According to Anthony et al. (2008), ocean acidification causes bleaching and loss in productivity in coral reef builders.

### Terrestrial sediments

At times, increased occurrence of fungal disease of marine invertebrates has been interpreted as a consequence of influx of terrestrial sediment (e.g. Garrison et al. 2003; Kellogg et al. 2004; Weir-Brush et al. 2004). In this context, dust storms are thought to be significant carriers and distributors of fungal spores that are introduced to the marine environment when the sediment is carried across the water (e.g. Kellogg et al. 2004; Kellogg and Griffin 2006).

### Light

Euendolithic fungi usually dominate as microborers of CaCO_3_ substrates in deep water environments, where light is reduced to a level that phototrophic microborers are excluded (e.g. Wisshak et al. 2005). In experiments in the laboratory and the field, marine fungi are suppressed by exposure to light or do not usually respond to light changes (e.g. Goldstein 1973; Curran 1980; Moree et al. 2014).

### Salinity

Different marine fungi grow in very different salinities but usually require comparatively stable conditions (Ritchie 1959; Alderman and Jones 1971a). In contrast to phototrophs, *O. implexa* was found in deeper, more uniform environments, where salinities did not much fluctuate (Wisshak et al. 2005). *O. implexa* can also occur in brackish water. Pirkova and Demenko (2008) described shell disease in *Crassostrea gigas*, the giant oyster, cultivated in the brackish Black Sea.

Many genera of fungi which are found in marine environments contain species and strains of species which differ greatly in salt tolerance. A good example of this is described by Gal-Hemed et al. (2011) in a study of salinity tolerance in species of *Trichoderma*.

A principle which has been described for marine fungi states that optimal growth patterns often rely on a direct relationship between temperature and salinity, i.e. best growth is reached at low salinities in low temperatures and in high salinities when temperatures are high (*Phoma* pattern; Ritchie 1959; Lorenz and Molitoris 1992). In shallow, stagnant waters hot weather with high evaporation rates could thus trigger fungal growth.

### Effects from human use and invasive species on marine molluscs

Marine molluscs have traditionally been exploited by humans not only for nutrition but also as providers of building materials, jewellery and ornamental shells (e.g. Alagarswami and Qasim 1973; Achuthankutty et al. 1979; Buestel et al. 2009). Fisheries quickly changed to mariculture in many countries, when wild stocks of fish became depleted (e.g. Alagarswami and Qasim 1973). A Brazilian study showed that cultured oysters were more likely to be diseased than wild conspecifics, which included shell disease (Da Silva et al. 2015), and in many locations, such cultures became afflicted. Responding to the demand, dwindling native cultures were sometimes replaced with similar species introduced from other countries – with varying success (e.g. Wolff et al. 2002; Buestel et al. 2009; Bromley et al. 2016).

However, introducing species into new ecosystems, where they have historically been absent, incurs an ecological risk and has often led to biological invasions (e.g. Bromley et al. 2016). Biological invasions can include both hosts and their parasites and have been widely recognised as potential major threats to marine biodiversity and in some cases, this has caused significant economic losses (Pimentel et al. 2001; Prenter et al. 2004). Euendoliths carried along with their hosts can either themselves infest the local mollusc populations that have not yet developed a resistance or interact with other microborers that were originally non-pathogenic. Either introduced or local fungi can suddenly become pathogenic and may cause changes to species composition and significant economic losses as well. Such species are often facultative pathogens.

Recently, facultative parasites have been implicated as key factors in the effects of invasions on species composition in marine ecosystems (Prenter et al. 2004). Four possibilities arise here. The decline in population sizes of commercially important species can be caused by (1) replacement by an invader which is better adapted to environmental conditions, (2) the spread of a newly emerging disease, (3) changes in environmental conditions or (4) a combination of these three factors (Prenter et al. 2004).

The interidal mussel *M. galloprovincialis* is a very successful species which invades mussel beds worldwide. In South Africa, for example, *Mytilus provincialis* has displaced the indigenous mussel *Aulacomya ater*, significantly reducing its population density, although it seems to coexist well with another indigenous species of mussel, *Perna perna* (Marquet et al. 2013). As the same authors found *M. provincialis* to be a carrier of microborers, it could represent a damaging vector dispersing marine diseases. A full understanding of the dynamics of biological invasions requires the consideration of the complex interactions between many abiotic and biotic factors.

## General comments/conclusions

Endoliths are important components of the marine calcium carbonate cycle, because they actively contribute to the biodegradation of shells of animals composed of calcium carbonate and calcareous geological substrates. They have been implicated as a causative agent of shell diseases in live corals, molluscs and other invertebrate animals which have shells composed of calcium carbonate (Golubic 1969; Kohlmeyer 1969; Che et al. 1996; Golubic et al. 2005; Zuykov et al. 2014).

Endolithic microorganisms have important roles as saprotrophs in bioerosion of many calcium carbonate substrates, as parasites on the production of commercially important animal species, regulate biodiversity in marine ecosystems, and they respond to environmental factors which are involved significant components of global climate change.

Heterotrophic endoliths can destroy the shells of animal species living in marine ecosystems or bioerode dead shells buried in the sediment (Kendrick et al. 1982; Raghukumar and Lande 1988). The dissolution of calcium carbonate in bioerosion causes the release of carbon dioxide into the marine environments, which increases acidification. Calcareous substrates contain large amounts of carbon. Therefore, heterotrophic endoliths are key players in the marine calcium carbonate cycle.

The total amount of global calcareous substrates in sediments in the ocean has not been accurately estimated. However, as carbon dioxide from bioerosion of calcium carbonate in the ocean eventually enters the atmosphere, large losses in calcareous substrates in carbon sinks would be expected to result in increased heat retention by the atmosphere, increasing global temperatures. If rising temperatures and acidity in the ocean increase the rate of growth of endolithic fungi, this could provide a positive feedback mechanism potentially accelerating the rate of climate change.

Many studies suggest that the prevalence of emerging infectious diseases is currently increasing in all ecosystems including coral reefs (e.g. Fisher et al. 2012; Burge et al. 2013). Heterotrophic endoliths including fungi are one group of parasites which can cause diseases in corals. Since fungal endoliths are present in both healthy and diseased corals, they are considered to be opportunist parasites which can cause disease but only under certain environmental conditions. Probably stress, depression of immune responses and global climate change factors are all important in triggering disease. None-the-less, if emerging infectious disease causes large losses in population sizes and in biodiversity, the lost carbon could enter the atmosphere as carbon dioxide produced by respiration as well.

Only three examples of documented diseases in corals are discussed in this review. We predict that more diseases will be identified in future research. Effective management of these diseases will be necessary in programmes designed to limit the rate of loss of coral populations and to re-establish coral population diversity in damaged reefs.

A number of species of Ascomycetes which produce clouds of conidia such as species of *Aspergillus, Penicillium, Cladosporium, Fusarium* and *Alternaria* also often synthesise secondary by-products which are toxic to animals including corals. Hayashi et al. (2016) provided evidence that toxic natural products produced by *A. sydowii* significantly inhibit the rate of photosynthesis in the zooxanthellae which provide food for gorgonian (soft) corals.

Opportunist marine fungal pathogens such as *A. sydowii* are ubiquitous in the open oceans, intertidal zones, marine sediments and animals which live in these ecosystems (Burge et al. 2013). The prevalence and consequences of these infections have increased in frequency and severity in recent decades (Soler-Hurtado et al. 2016).

The effect of environmental conditions on stages of the life cycle has not been carefully studied in most isolates of ascomycetes from marine environments. Many isolates of ascomycetes can form sexual or teleomorphic stages in the laboratory (Pitt and Hocking 2009) but this stage has not yet been observed during growth on substrates in sea water. Ability to penetrate calcareous substrates and pathogenicity has not been studied in most ascomycete genera either.

We expect there to be large genetic variability in populations of species of pathogens occurring naturally in marine ecosystems, but data to support this conclusion are not yet available for most species. However, Varga and Tóth (2003) reported genetic variability in *Aspergillus fumagatus*, a pathogen similar to *A. sydowii*.

It is also possible that fungi play a positive role in some animal/fungal associations such as in symbiotic relationships. If so, none has so far been investigated in detail and this research topic needs to be further investigated in future.

Coral reef ecosystems are losing biodiversity rapidly at present with improper management and climate change (Hoegh-Guldberg 1999). Fungi are important components of the coral reef ecosystem (Gleason et al. 2017) and must be included in ecological studies.
